# Identification to the species level of *Lactobacillus *isolated in probiotic prospecting studies of human, animal or food origin by 16S-23S rRNA restriction profiling

**DOI:** 10.1186/1471-2180-5-15

**Published:** 2005-03-23

**Authors:** João Luiz S Moreira, Rodrigo M Mota, Maria F Horta, Santuza MR Teixeira, Elisabeth Neumann, Jacques R Nicoli, Álvaro C Nunes

**Affiliations:** 1 Departamento de Biologia Geral, Instituto de Ciências Biológicas, Universidade Federal de Minas Gerais, Av. Antônio Carlos 6627, 31.270-901, Belo Horizonte, MG, Brazil; 2 Departamento de Bioquímica e Imunologia, Instituto de Ciências Biológicas, Universidade Federal de Minas Gerais, Av. Antônio Carlos 6627, 31.270-901, Belo Horizonte, MG, Brazil; 3 Departamento de Microbiologia, Instituto de Ciências Biológicas, Universidade Federal de Minas Gerais, Av. Antônio Carlos 6627, 31.270-901, Belo Horizonte, MG, Brazil; 4 Centro Universitário Newton Paiva, Rua Goitacases 1762, 30.190-052, Belo Horizonte, MG, Brazil

## Abstract

**Background:**

The accurate identification of *Lactobacillus *and other co-isolated bacteria during microbial ecological studies of ecosystems such as the human or animal intestinal tracts and food products is a hard task by phenotypic methods requiring additional tests such as protein and/or lipids profiling.

**Results:**

Bacteria isolated in different probiotic prospecting studies, using de Man, Rogosa and Sharpe medium (MRS), were typed at species level by PCR amplification of 16S-23S rRNA intergenic spacers using universal primers that anneal within 16S and 23S genes, followed by restriction digestion analyses of PCR products. The set of enzymes chosen differentiates most species of *Lactobacillus *genus and also co-isolated bacteria such as *Enterococcus*, *Streptococcus*, *Weissella*, *Staphylococcus*, and *Escherichia *species. The in silico predictions of restriction patterns generated by the *Lactobacillus *shorter spacers digested with 11 restriction enzymes with 6 bp specificities allowed us to distinguish almost all isolates at the species level but not at the subspecies one. Simultaneous theoretical digestions of the three spacers (long, medium and short) with the same set of enzymes provided more complex patterns and allowed us to distinguish the species without purifying and cloning of PCR products.

**Conclusion:**

*Lactobacillus *isolates and several other strains of bacteria co-isolated on MRS medium from gastrointestinal ecosystem and fermented food products could be identified using DNA fingerprints generated by restriction endonucleases. The methodology based on amplified ribosomal DNA restriction analysis (ARDRA) is easier, faster and more accurate than the current methodologies based on fermentation profiles, used in most laboratories for the purpose of identification of these bacteria in different prospecting studies.

## Background

Probiotics are food or preparations containing live microorganisms, traditionally regarded as safe for human use. When ingested in sufficient numbers, probiotics are believed to play an important role in the control of host intestinal microbiota and maintenance of its normal state [2]. Microbes that are frequently isolated from human and animal intestines and selected as probiotics, include species of the genera *Lactobacillus*, *Bifidobacterium*, and *Enterococcus*. However, some other lactic acid producing bacteria that do not normally inhabit the intestinal tract are also sometimes used as probiotics. Most of these bacteria are used as starters in dairy products and include *Streptococcus*, *Lactococcus*, *Leuconostoc *and *Pediococcus *species. Yet, as different types of lactic acid producing bacteria can affect the human intestinal microenvironment in different ways, it is important to identify which microorganisms are present in a microbial ecosystem and which species are most likely to have the potential protective effects.

The precise identification of these bacteria to the species level is not an easy task. As pointed out by Tannock et al. [10], the identification of *Lactobacillus *isolates by phenotypic methods is difficult because it requires, in several cases, determination of bacterial properties beyond those of the common fermentation tests. In general, about 17 phenotypic tests are required to identify an isolate of *Lactobacillus *accurately at the species level. Most commercial kits, frequently used in laboratory routine, are able to precisely identify only 30% of vaginal and intestinal isolates from humans [8]. The establishment of a simple and fast identification method is therefore required in order to be able to deal with the large numbers of *Lactobacillus *isolates that can be obtained during microbial ecological studies of ecosystems such as the human or animal intestinal tracts and food products.

In addition to *Lactobacillus*, other lactic acid bacteria (LAB) and several other groups of bacteria are found in the same habitats during ecological studies of microbiota succession. Methodological procedures to handle such a high number of isolates should be brief and precise to avoid inconclusive results. Due to the growing interest in using bacteria as probiotics or starters in dairy products, the identification of these microorganisms at species level is becoming more and more required. DNA sequencing of PCR amplified conserved genes such as rRNA ones is the most used methodology for typing microorganisms. However, it requires special equipments and dealing with hundred of different isolates is expensive.

The aim of this study was to develop an easy and fast procedure to accurately identify to the species level most of *Lactobacillus *type and reference strains and new strains isolated from different ecological studies. In these different prospecting studies, we are looking for new *Lactobacillus *strains to be used as 1) a vehicle to delivery chimerical transgenes to immunize broiler chicks against coccidiosis, 2) antagonizers of pathogenic bacteria during cheese and cassava starch processing to improve food safety and quality, and 3) live medicine against women vaginosis and vaginitis. Therefore, the correct typing of these new strains is critical.

Analysis of the number and size of the intergenic regions between 16S and 23S genes showed that they vary among *Lactobacillus *and others bacteria allowing genera discrimination (unpublished results). Other groups have published methodologies based on the fingerprinting of the variable region of the 16S rRNA [14]. We choose to amplify the 16S-23S rRNA intergenic spacers because this region allowed us to discriminate a larger number of species of *Lactobacillus*. Typical patterns of *Lactobacillus *amplicons presented three bands, corresponding to the short, medium and long spacer regions, similar to those seen in *Pediococcus*, *Carnobacterium*, *Leuconostoc *and *Weissella *species [4]. These differences in size are due to the insertion of a tRNA-Ala gene within the medium spacer or tRNA-Ala and tRNA-Ile genes within the long spacer. Bifidobacteria, enterococci, streptococci and staphylococci presented patterns with a unique band or two bands with different sizes also due insertion of tRNA genes. The methodology proposed here can replace fermentation tests for characterization of a number of clinical, health food, and laboratory isolates through PCR amplification of 16S-23S rRNA intergenic spacers followed by digestion with a set of restriction enzymes with 6 bases recognition sequences.

## Results

The *in silico *predictions of restriction patterns of 16S-23S rRNA short intergenic spacer of selected *Lactobacillus *species and of other related bacteria frequently isolated from the same sources of *Lactobacillus *are shown in Tables 1 and 2, respectively. Although for most bacteria different patterns can be predicted, some species present identical patterns. In this case, however they can be differentiated by the sizes of the two amplified bands (*E. faecium *amplicons are almost 100 bp larger than *E. faecalis *amplicons).

**Figure 1:**
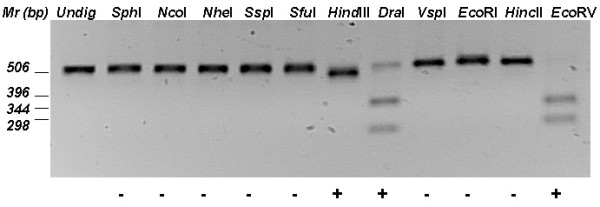
**Restriction digestion of 16S-23S short intergenic spacer of *Lactobacillus rhamnosus *GG. **PCR amplification of rRNA spacer regions was done and the shorter amplicon was excised from an agarose gel after electrophoresis at 100 V for 45 min. Gel purified DNA was cleaved with 11 enzymes and fragments separated by electrophoresis as before. Plus and minus signs mean positive or negative cleavage of PCR amplicon. GibcoBRL 1 kb DNA ladder was used for sizing DNA fragments and molecular masses are shown at right in bp.

Fig. 1 shows a typical gel of the short intergenic spacer of *Lact. rhamnosus *GG digested with 11 restriction enzymes, in which DNA cleavage was observed with *Hind*III, *Dra*I and *Eco*RV. The uncut DNA at *Dra*I lane may reflect some polymorphism of the short spacer nucleotide sequences in different operon units. The patterns obtained with digestion of the 16S-23S rRNA short spacer alone could distinguish most of *Lactobacillus *at species level but, in some cases, such as among *Lact. acidophilus*, *Lact. helveticus *and *Lact. amylovorus*, they could only be grouped as 'acidophilus' clad. The separation of acidophilus group species could be achieved by nucleotide sequencing or by digestion of the others two intergenic spacers in order to search for more complex restriction patterns.

**Figure 2:**
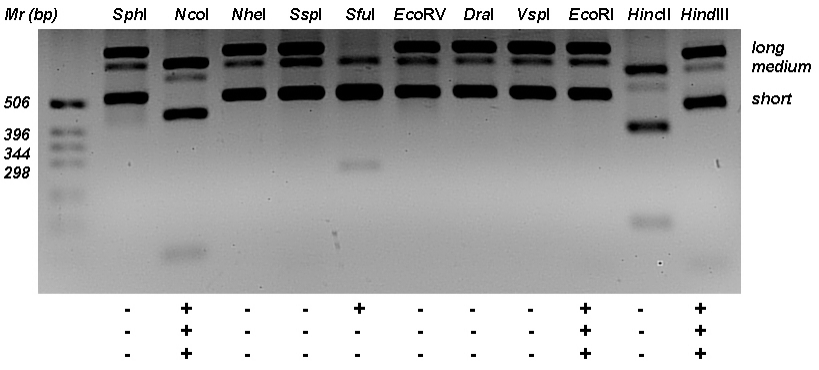
**Restriction digestion of the three 16S-23S intergenic spacers of *Lactobacillus acidophilus *ATCC 4356. **PCR amplification of rRNA spacer regions was done and amplicons were cleaved with 11 restriction enzymes and fragments separated by agarose gel electrophoresis at 100 V for 45 min. Plus and minus signs mean positive or negative cleavage of long, medium or short PCR amplicon, respectively. GibcoBRL 1 kb DNA ladder was used for sizing DNA fragments and molecular masses are shown at right in bp.

Fig. 2 shows a gel with simultaneous digestion of the three 16S-23S rRNA intergenic spacers of *Lact. acidophilus *ATCC 4356 with the same set of enzymes used to digest the short spacers. The three spacers of *Lact. acidophilus *were cut with *Nco*I, *Eco*RI and *Hind*III enzymes, but only the long spacer was additionally digested with *Sfu*I. Looking at sequences deposited at GenBank, we observed that the long spacer of *Lact. helveticus *have a site for *Sfu*I, but unlike *Lact. acidophilus*, it can also be cut with *Dra*I, a feature that can be used to differentiate these species However, this theoretical finding has not yet been validated through PCR-ARDRA. The simultaneous digestion of the three intergenic spacers could therefore improve the distinctiveness of the methodology proposed here, allowing the identification of very closely related species.

We tested the methodology of 16S-23S rRNA amplification and restriction digestion in a set of isolates from chicken gastrointestinal tract, human sources, and food. Table 3 shows the digestion patterns of forty-three isolates and their identification at the species level. All bacteria except *Lact. gasseri*, *Lact. johnsonii *and *Lact. jensenii *isolates (Reg 24, 25, 26 and 32 from chicken faeces, and PM1, 2 and 3 from human vagina) could be typed based on the restriction digestion pattern of the short 16S-23S rRNA intergenic region obtained with the 11 enzymes tested. The short spacers of these three *Lactobacillus *species could be cleaved only with *Eco*RV enzyme, and to distinguish between each species it was necessary to include the digestion profile of the medium and long spacers cleaved with *Hind*III, *Pvu*II and *Bgl*II. *Lact. gasseri *spacers were not cleaved with any of these three enzymes, while *Lact. johnsonii *long spacer could be cleaved with *Hind*III and *Pvu*II, and *Lact. jensenii *long spacer could be cleaved also with *Bgl*II. To validate the PCR-ARDRA identification, the forty-three short amplicons from those *Lactobacillus *isolates were cloned and sequenced. The level of identity between aligned sequences was above 98% for all isolates.

Searching nucleotide databases we found deposited sequences of long and medium spacers of some *Lactobacillus *species and also of most species of *Carnobacterium *and *Weissella*, two closely related genera. Table 4 exemplifies how simultaneous cleavage of the three spacers of some *Weissella *species can improve the methodology of PCR-ARDRA proposed here.

## Discussion

The precise identification of microorganisms presenting economical interest is a prerequisite to select new strains among several bacteria isolates. The lactic-acid producing bacteria associated with foods or used as probiotics include species of the genera *Lactobacillus*, *Carnobacterium*, *Enterococcus*, *Lactococcus*, *Leuconostoc*, *Oenococcus*, *Pediococcus*, *Streptococcus*, *Tetragenococcus*, *Vagococcus *and *Weissella*. The genus *Lactobacillus *is heterogeneous, presenting over 60 species showing G+C content ranging from 33 to 55 % [9]. This high heterogeneity is reflected by phenotypic tests of *Lactobacillus *isolates from diverse ecosystems, which show intermediate sugar fermentation profiles that hamper a more precise typing at species level. Although several DNA-based procedures are now available, most of them require special techniques or laboratory devices and can also be time-consuming, which makes identification of a hundred isolates a fastidious job.

Besides their use as food microorganisms, the use of LAB as bacterial systems to express heterologous proteins or as vehicles to carry immunizing antigens after genetic modification [7] is becoming a promising issue due to the nutritional benefits and almost null pathogenicity of these bacteria. The successful expression of heterologous proteins in LAB depends on the promoter compatibility between the species or strains used as vector or hosts. As pointed out by Pouwels et al. [6], the control of transcription and translation may differ greatly between two *Lactobacillus *species, implying that the knowledge generated for one organism may not simply be transferred to another. Genes that are efficiently expressed in one *Lactobacillus *species are not necessarily expressed in other species, or are expressed with different efficiency and/or with a different regulatory mechanism. Therefore, the correct typing of new isolates with probiotic properties is crucial for the development of a successful oral vaccine, such as the one we are intending to make against avian coccidiosis delivered by genetically engineered *Lactobacillus*.

As pointed out in Table 1, a large number of *Lactobacillus *species can be typed effectively on the basis of the restriction patterns of their 16S-23S intergenic regions, after a selection step using the MRS media. This method constitutes a specific and reproducible way to identify LAB bacteria at the species level. In addition, most of other bacteria genera co-isolated with *Lactobacillus *in these prospective studies could also be accurately identified through 16S-23S spacer PCR-ARDRA (Table 2). Forty-three new strains of *Lactobacillus *obtained from animals, human or foods could be easily identified by this methodology, without need of gel purification and cloning (Table 3). The differences in intensities of the bands corresponding to the three 16S-23S rRNA spacers (Fig. 2) is probably due the number of rRNA operons with each spacer. This can also explain the partial digestions of shorter spacers observed occasionally such as the one observe in *L. rhamnosus *GG digested with *Dra*I (Fig. 1). The simultaneous digestion of all PCR amplicons corresponding to the three *Lactobacillus *16S-23S rRNA spacers showed that these regions are very polymorphic and suitable for distinguishing them at species level. Similar polymorphisms were identified in DNA sequences deposited at GenBank of the three 16S-23S rRNA spacers of *Weissella *species (Table 4) and shall probably occur in *Leuconostoc*, *Carnobacterium*, *Pediococcus *and other genera closely related to *Lactobacillus*.

Although we have initially employed the short spacers to distinguish *Lactobacillus *species as proposed by Tannock et al. [10], the main improvement described here is a typing protocol that avoids gel purification and nucleotide sequencing of the amplicons. The restriction digestion of the three spacers showed that a very good discrimination of several *Lactobacillus *and other bacteria could be achieved by direct digestion of PCR products followed by a single gel electrophoresis to verify the restriction pattern of the amplicons. Our procedure is faster and less expensive than the method originally proposed by Tannock's group. However, gel electrophoresis was still necessary to visualize the restricted fragments. To overcome that, we choose restriction enzyme with 6 bases recognition sites, which cut less frequently and, thus, generate simple restriction digestion patterns. Because our goal is to avoid the electrophoresis step, we are currently working on a protocol that could adapt the PCR-ARDRA into a more straightforward method such as a PCR-ELISA. With this protocol, instead of using gel electrophoresis, the positive result of a digestion reaction could be detected colorimetrically, after incorporation of labelled nucleotides, performed simultaneously with the cleavage reaction. In this way, the whole procedure can be carried out in any laboratory equipped with a PCR machine.

## Conclusion

Forty-three *Lactobacillus *isolated and several strains of other bacteria co-isolated in different prospecting studies to select new bacteria with probiotic features could be processed and identified using the proposed approach. The methodology, based on the small 16S-23S rRNA spacer ARDRA is accurate but needs gel electrophoresis to visualize the digested products. In order to validate the restriction pattern obtained, we also purified and sequenced the amplicons corresponding to the short spacer. The amplification and the restriction digestion of the three spacer regions allowed us to discriminate the *Lactobacillus *species without the need of gel excision and purification but still requires the electrophoresis step to visualize the digested products. These results represent a first step in the development of an easier and faster protocol that may allow us to detect changes in the restriction patterns using a PCR-ELISA kit.

## Methods

### Bacterial strains and growth conditions

The following bacteria were used in this study to validate the theoretical restriction patterns of Tables 1 and 2: *Lactobacillus acidophilus *ATCC 4356T, *Lact. brevis *ATCC 367, *Lact. casei *ATCC 393T, *Lact. delbrueckii subsp. lactis *ATCC 7830, *Lact. fermentum *ATCC 9338, *Lact. plantarum *ATCC 8014, *Lact. plantarum *NCDO 1193, *Lact. reuteri *DSM 20016T, *Lact. rhamnosus *GG ATCC 53103, Lact. *sakei *NCFB 3714, *Bacteroides fragilis *ATCC 25285T, *Bifidobacterium longum *Bb46 and *Bif. lactis *Bb12 (Christian Hansen Laboratory, Horsholm, Denmark), cheese isolates of *Enterococcus faecalis*, *Staphylococcus aureus*, *Weissella paramesenteroides*, *Lactococcus lactis*, *Streptococcus thermophilus *typed by nucleotide sequencing, *Escherichia coli *XL1-Blue (Stratagene), and *Pediococcus pentosaceus *ATCC 33314. We are not able to validate the theoretical restriction patterns of several species of *Lactobacillus *or other bacteria and for most species several sequences were available at GenBank deposited by different research groups. *Oenococcus*, *Carnobacterium*, *Leuconostoc *and *Propionibacterium *specimens were not typed in this work but theoretical digestion patterns are also presented in Table 2.

Forty-three *Lactobacillus *isolates from chicken intestines, human vagina or faeces, Brazilian 'coalho' cheese, and cassava starch sour fermentation from collection of the Department of Microbiology, Universidade Federal de Minas Gerais, were typed in this study by PCR-ARDRA as proposed and verified by nucleotide sequencing to confirm species identification. All lactic acid bacteria were stored at -80°C in de Man Rogosa Sharpe (MRS) broth (Difco, Detroit, MI, USA) with 30% glycerol. Before experimental use, cultures were grown anaerobically in MRS medium at 37°C and subcultured twice in MRS. *E. coli *was grown aerobically in Luria-Bertani medium and *Bacteroides *anaerobically in BHI medium.

### DNA extraction

Chromosomal DNA was isolated from overnight cultures of *Lactobacillus *sp in 10 ml MRS broth. After washing the cells with 10 ml of deionised water, the pellet was resuspended in 1 ml of 5 M LiCl and incubated for 1 h under constant shaking. Cells were washed once more with 1 ml of deionised water and the pellet was resuspended in 1 ml of protoplasting buffer (25 mM sucrose, 50 mM Tris HCl pH 8.0, 10 mM EDTA, 10 mg of lysozyme ml^-1^, 100 μg of RNaseA ml^-1^). After incubation for 1 h at 37°C and centrifugation at maximum speed in a microcentrifuge for 5 min, the pellet was resuspended in 500 μl of protoplasting buffer without sucrose and lysozyme, and 100 μl of 10% sodium dodecyl sulfate were added to allow cells lysis. The mixture was extracted once with phenol, with phenol-chloroform-isoamyl alcohol (25:24:1) and with chloroform-isoamyl alcohol (24:1). After isopropanol precipitation the DNA was dissolved in 100 μl of TE buffer.

### PCR amplification of 16S-23S rDNA intergenic spacer

The 16S-23S intergenic spacer region amplification was carried out according to Tilsala-Timisjarvi and Alatossava [12] using the primer 16-1A, 5'-GAATCGCTAGTAATCG-3', corresponding to nucleotides 1361 to 1380 of the 16S rRNA gene according to *Lactobacillus casei *numbering, and the primer 23-1B, 5'-GGGTTCCCCCATTCGGA-3', corresponding to nucleotides 123 to 113 of the 23S rRNA of *L. casei*. The reaction mixture (50 μl) contained 10 pmol of each primer, 0.2 mM of each deoxyribonucleotide triphosphate, reaction buffer with 1.5 mM MgCl_2_, 5 U of *Taq *DNA polymerase (Phoneutria Biotecnologia & Serviços, Brazil), and of 10 ng of template DNA. The amplification program used was: 94°C for 2 min, 35 cycles of 94°C for 30 s, 55°C for 1 min, 72°C for 1 min, and 72°C for 10 min. PCR products were electrophoresed in a 1.4% agarose gel and visualized by UV transillumination after ethidium bromide staining.

### Restriction digestion

The 16S-23S short intergenic spacer regions of the *Lactobacillus *bacteria were digested by a set of 11 enzymes chosen after compilation of nucleotide sequences already deposited at GenBank and theoretical restriction digestion with Webcutter 2.0 tool (Max Heiman, ). *Sph*I, *Nco*I and *Nhe*I enzymes cleave inside 16S gene, *Ssp*I, *Sfu*I, *Dra*I, *Vsp*I, *Hinc*II and *Eco*RI enzymes cleave inside the intergenic region, and *Avr*II and *Hind*III enzymes cleave inside 23S gene. *Eco*RV enzyme cleaves inside spacer region in *Lact. casei *group and in the 23S gene in *Lact. acidophilus *group. All restriction enzymes were purchased from Promega Corporation (Madison, WI, USA).

### Cloning of the PCR-amplified products

*Lactobacillus *species frequently contain three 16S-23S intergenic regions (long, medium and short spacers), in which case the smallest PCR amplicon (about 500 to 600 bp) was excised from the gel and purified with the Concert™ Rapid Gel Extraction System according to the manufacturer's instructions (Invitrogen Life Technologies, Carlsbad, CA, USA). PCR products were cleaned-up using the Concert™ Rapid PCR Purification System and cloned in *E. coli *XL1-Blue into pCR2.1-TOPO vector using the Invitrogen TOPO TA cloning kit (Invitrogen). PCR was performed on cell lysates of ampicillin-resistant transformants using M13 specific primers to confirm the size of the inserts. Plasmids containing a unique insert of the appropriate size were purified by the GFX™ Micro Plasmid Prep kit (Amersham Biosciences, Piscataway, NJ, USA) and were subjected to DNA sequence analysis to validate PCR-ARDRA species identification.

### Sequencing and sequence analysis

DNA sequencing of PCR amplicons was carried out at the Núcleo de Análise de Genoma e Expressão Gênica (NAGE), Universidade Federal de Minas Gerais, using a DYEnamic™ ET Dye Terminator Cycle Sequencing kit for MegaBACE™ DNA Analysis Systems (Amersham Biosciences, USA) in combination with a MegaBACE™ 1000 automated sequencing system. Both polynucleotide strands of the cloned DNA were sequenced, using M13 forward and reverse primers. The short intergenic spacer region sequences obtained by these methods were compared to sequences from type culture and other *Lactobacillus *strains held in GenBank DNA database using the BLAST algorithm [1] to validate the theoretical restriction patterns used for species identification by PCR-ARDRA.

## Authors' contributions

JLSM and RMM carried out all the experiments used for the molecular biology studies: DNA extraction, PCR amplification, gel electrophoresis, restriction digestion. MFH and SMRT participated in the sequence alignment, the discussion of the results, and the manuscript draft. EN and JRN participated in the study design. ACN conceived and designed the study, and coordination and helped to draft the manuscript. All authors read and approved the final manuscript.

## Figures and Tables

**Table 1:** Theoretical patterns of restriction digestion of short 16S-23S intergenic spacer in different species of *Lactobacillus*

	*Sph*I	*Nco*I	*Nhe*I	*Ssp*I	*Sfu*I	*Eco*RV	*Dra*I	*Vsp*I	*Hinc*II	*Eco*RI	*Hind*III
*Lact. delbrueckii*	-^a^	+^a^	-	-	+	-	-	-	-	-	-
*Lact. amylolyticus*	-	+	-	-	+	-	-	-	-	-	+
*Lact. acidophilus*	-	+	-	-	-	-	-	-	-	+	+
*Lact. amylovorus*	-	+	-	-	-	-	-	-	-	+	+
*Lact. helveticus*	-	+	-	-	-	-	-	-	-	+	+
*Lact. crispatus*	-	+	-	-	-	+	-	-	-	+	+
*Lact. perolens*	-	-	-	-	-	+	-	-	-	-	+
*Lact. gasseri*	-	-	-	-	-	+	-	-	-	-	-
*Lact. johnsonii*	-	-	-	-	-	+	-	-	-	-	-
*Lact. jensenii*	-	-	-	-	-	+	-	-	-	-	-
*Lact. reuterii*	+	+	-	-	-	-	-	+	-	-	-
*Lact. vaginalis*	+	+	-	-	-	-	-	++^b^	-	-	+
*Lact. brevis*	+	-	-	-	-	-	-	+	-	-	+
*Lact. sakei*	+	-	-	-	-	-	++	-	-	-	+
*Lact. casei*	-	-	-	-	-	+	+	+	-	-	+
*Lact. rhamnosus*	-	-	-	-	-	+	+	-	-	-	+
*Lact. salivarius*	-	-	-	+	-	-	++	+	+	-	-
*Lact. ruminis*	-	-	-	+	+	-	+	-	-	-	-
*Lact. plantarum a*	+	-	-	+	-^c^	-	-	+	+	-	-
*Lact. plantarum b*	+	-	-	+	+^c^	-	-	+	+	-	-
*Lact. fermentum*	+	-	-	-	-	-	-	+	-	-	-

^a^Plus and minus signals represent positive and negative restriction digestion, respectively

^b^Two or more restriction sites found into spacer region

^c^Polymorphic site present in some strains of *Lact. plantarum *and absent in others

**Table 2:** Theoretical patterns of restriction digestion of short 16S-23S intergenic spacer in different species of *Bacteroides*, *Bifidobacterium*, *Carnobacterium*, *Enterococcus*, *Escherichia*, *Lactococcus*, *Leuconostoc*, *Oenococcus*, *Pediococcus*, *Propionibacterium*, *Staphylococcus*, *Streptococcus*, and *Weissella*

	*Sph*I	*Nco*I	*Nhe*I	*Ssp*I	*Sfu*I	*Eco*RV	*Dra*I	*Vsp*I	*Hinc*II	*Eco*RI	*Avr*II	*Hind*III
*Bact. fragilis*	-	-	-	-	+	-	-	-	+	-	-	-
*B. longum*	-	-	-	-	+	-	-	-	-	+	-	-
*B. lactis*	-	-	-	-	-	-	-	-	-	-	-	-
*C. piscicola*	-	-	-	+	-	-	++^a^	-	-	-	-	+
*C. gallinarum*	-	-	-	-	-	-	++	-	-	+	-	+
*C. mobile*	-	-	-	-	-	-	-	-	-	+	-	-
*Ent. faecalis*	-	-	-	+	-	-	-	-	-	-	-	-
*Ent. faecium*	-	-	-	+	-	-	-	-	-	-	-	-
*Ent. raffinosus*	-	-	-	+	-	-	-	-	-	-	-	+
*E. coli*	-	+	-	-	+	-	+	-	-	-	+	-
*L. lactis*	-	-	-	-	-	-	+	-	-	-	+	-
*Leuc. mesenteroides*	-	-	+	-	-	-	++	-	-	-	-	+
*O. oeni*	-	-	-	-	+	-	+	-	-	-	-	+
*Ped. acidilacitii*	+	-	+	-	-	-	+	++	-	-	-	+
*Ped. parvulus*	+	-	+	-	-	-	+	-	-	-	-	+
*P. freudenreichii*	-	-	-	-	-	-	-	-	-	-	+	-
*Staph. aureus*	+	-	+	+	+	-	+++	+	+	+	-	++
*Strep. thermophilus*	-	-	-	+	-	-	-	-	-	-	-	-
*W. confusa*	-	-	-	+	-	-	+++	-	-	-	-	+
*W. paramesenteroides*	-	-	-	-	-	-	+++	-	-	-	-	-

^a^Two or more restriction sites found into spacer region

**Table 3:** Patterns of restriction digestion of short 16S-23S intergenic spacer of lactobacilli and other bacteria isolated from different sources

Isolate	*Sph*I	*Nco*I	*Nhe*I	*Ssp*I	*Sfu*I	*Eco*RV	*Dra*I	*Vsp*I	*Hinc*II	*Eco*RI	*Hind*III	Identification
Chicken gastrointestinal tract												
*F 5.2* *F 5.3*	+	-	-	+	+	-	-	+	+	-	-	*Lact. plantarum *b
*F 5.7*	-	+	-	-	-	+	-	-	-	+	+	*Lact. crispatus*
*Reg 24* *25 26*	-	-	-	-	-	+	-	-	-	-	-	*Lact. johnsonii*^a^
*Reg 32*	-	-	-	-	-	+	-	-	-	-	-	*Lact. gasseri*^a^
*Reg 34*	+	+	-	-	-	-	-	+	-	-	-	*Lact. reuteri*
*38 90* *110*	+	+	-	-	-	-	-	+	-	-	+	*Lact. vaginalis*
*98, 108*	-	-	-	-	-	+	+	-	-	-	+	*Lact. rhamnosus*
*115*	-	+	-	-	-	-	-	-	-	+	+	*Lact. acidophilus*
'Coalho' cheese												
*AB2*	-	-	-	-	-	+	+	+	-	-	+	*Lact. casei*
*AB3*	+	-	-	-	-	-	-	+	-	-	-	*Lact. fermentum*
*AQ1*	-	-	-	-	-	+	+	-	-	-	+	*Lact. rhamnosus*
*LV3*	-	+	-	-	-	-	-	-	-	+	+	*Lact. acidophilus*
*CM3*	-	-	-	+	-	-	+	-	-	-	+	*W. confusa*
*AB1 BL1* *SM4*	-	-	-	+	-	-	-	-	-	-	-	*Strep. thermophilus*
*CM2M17*	-	-	-	-	-	-	+	-	-	-	-	*L. lactis*
*AQ2* *CM4* *LV1*	-	-	-	+	-	-	-	-	-	-	-	*Ent. faecalis*^b^
*AQ2AT1*	+	-	+	+	+	-	+	+	+	+	+	*Staph. aureus*
Cassava starch sour fermentation												
*P16.01* *P25.01*	+	-	-	-	-	-	-	+	-	-	-	*Lact. fermentum*
*P24.03* *P25.04*	+	-	-	+	+	-	-	+	+	-	-	*Lact. plantarum *b
*P24.01* *P29.01*	+	-	-	+	-	-	-	+	+	-	-	*Lact. plantarum *a
*P10.06*	+	-	-	-	-	-	-	+	-	-	+	*Lact. brevis*
*P10.02*	-	-	-	+	-	-	+	+	+	-	-	*Lact. salivarius*
*P18.04*	-	-	-	-	-	+	-	-	-	-	+	*Lact. perolens*
Human sources												
*UFV* *H2b20*	-	+	-	-	+	-	-	-	-	-	-	*Lact. delbrueckii*
*117*	-	-	-	-	-	+	+	-	-	-	+	*Lact. rhamnosus*
*A15* *A91*	-	-	-	+	+	-	+	-	-	-	-	*Lact. ruminis*
*PM1*	-	-	-	-	-	+	-	-	-	-	-	*Lact. johnsonii*^a^
*PM2* *PM3*	-	-	-	-	-	+	-	-	-	-	-	*Lact. jensenii*^a^

^a^Species identified by additional restriction digestion with *Hind*III, *Bgl*II and *Pvu*II of long spacer regions

^b^Species identified by amplicons sizes

**Table 4:** Theoretical patterns of restriction digestion of the three 16S-23S intergenic spacers in some species of *Weissella*

	*Sph*I	*Nco*I	*Nhe*I	*Ssp*I	*Sfu*I	*Eco*RV	*Dra*I	*Vsp*I	*Hinc*II	*Eco*RI	*Hind*III
*W. viridescens*											
long^a^	-	-	-	-	-	-	+	+	-	-	+
medium^b^	-	+	-	-	-	-	+	+	-	-	+
short	-	-	-	-	-	-	+	+	-	-	+
*W. kimchii*											
long	-	-	-	+	-	-	+	-	+	-	+
medium	-	+	-	+	-	-	+	-	+	-	+
short^c^											
*W. kandleri*											
long^c^											
medium	-	-	-	+	-	-	+	-	+	-	+
short	-	-	-	+	-	-	+	+	-	-	+
*W. confusa*											
long	-	-	-	+	-	-	+	-	-	-	+
medium	-	-	-	+	-	-	+	-	-	-	+
short	-	-	-	+	-	-	+	-	-	-	+
*W. minor*											
long	-	-	-	+	-	-	+	-	-	-	+
medium	-	-	-	+	-	-	+	-	-	-	+
short^c^											
*W. thailandensis*											
long	-	-	-	-	+	-	+	-	-	-	+
medium	-	-	-	-	+	-	+	-	-	+	+
short	-	-	-	-	-	-	+	-	-	+	+
*W. paramesenteroides*											
long	-	-	-	-	+	-	+	-	-	-	-
medium	-	-	-	-	+	-	+	-	-	+	-
short	-	-	-	-	-	-	+	-	-	-	-

^a^tRNA-Ile and tRNA-Ala genes inserted

^b^tRNA-Ala gene inserted

^c^Sequences not found in GenBank database
